# A New Method for Analyzing Aero-Optical Effects with Transient Simulation

**DOI:** 10.3390/s21062199

**Published:** 2021-03-21

**Authors:** Bo Yang, Zichen Fan, He Yu, Haidong Hu, Zhaohua Yang

**Affiliations:** 1 School of Astronautics, Beihang University, Beijng 100191, China; yangbo@buaa.edu.cn (B.Y.); AlbertYH@buaa.edu.cn (H.Y.); 2 The Science and Technology on Space Intelligent Control Laboratory of China, Beijing 100190, China; haidong_2005@163.com; 3 School of Instrumentation and Optoelectronic Engineering, Beihang University, Beijng 100191, China; yangzh@buaa.edu.cn

**Keywords:** micro mechanism of aero-optical effects, micro-scale density structure, photon energy scattering, scaling model of distorted wavefront, artificial vortex structure, star sensor of hypersonic vehicle

## Abstract

Aero-optical effects reduce the accuracy of optical sensors on high-speed aircraft. Current research usually focuses on light refraction caused by large-scale density structures in turbulence. A method for analyzing photon energy scattering caused by micro-scale structures is proposed in this paper, which can explain the macro image distortion caused by moving molecules in inhomogeneous airflow. Quantitative analysis of the propagation equation indicates that micro-scale structures may contribute more to the wavefront distortion than the widely considered large-scale structures. To analyze the micro mechanism of aero-optical effects, a transient simulator is designed based on the scaling model of transient distorted wavefronts and the artificial vortex structure. The simulation results demonstrate that correct aero-optical phenomena can be obtained from the micro mechanism of photon energy scattering. Examples of using the transient simulator to optimize the parameters of the star sensor on a hypersonic vehicle are provided. The proposed analysis method for micro-scale structures provides a new idea for studying the aero-optical effects.

## 1. Introduction

With the rapid development of hypersonic vehicles, the measurement errors of optical sensors caused by aero-optical effects have attracted research attention [1,2,3,4,5]. The transmission of light through a high-speed flow field is disturbed by the inhomogeneous and time-varying density field, which seriously reduces the imaging quality of the optical sensor and causes image blurring, deviation, and jitter. Areas that suffer from aero-optical effects include infrared seeker imaging [6], airborne laser communication [7], airborne directed energy weapons [8], and celestial navigation [1,9]. The radar seeker of a ballistic missile is also disturbed by inhomogeneous airflow in the terminal guidance phase, which leads to impact errors.

The cause of aero-optical effects is usually explained as light refraction, and large-scale density structures in turbulence are considered the main contributor [10,11]. However, light refraction is a macro description that cannot accurately reveal the micro mechanism of aero-optical effects because the influence from the scattering of micro-scale structures, which is close to the dissipative scale, is not negligible compared with the refraction caused by large-scale structures [12,13]. Light refraction is the superposition result of the scattering caused by many micro-scale structures. When the medium is homogeneous or locally homogeneous, the scattering can be replaced by the refraction. However, the high-speed flow field contains plenty of unstable turbulent structures, whose density distribution changes rapidly in both the time and space domains. If we only use the present macro refraction mechanism to describe the transmission of light, the distortion is underestimated. A detailed analysis is presented in Section 2.

Due to the high cost of flight and wind tunnel experiments, the analysis of aero-optical effects in the preliminary design stage of high-speed aircraft depends on simulation. Computational fluid dynamics (CFD) is a widely used simulation method to compute the density distribution of large-scale structures, and then the distribution of the refractive index is further obtained by the Gladstone-Dale law [14,15]. However, due to the limitation of mesh resolution, the results of CFD cannot be directly used to analyze the scattering caused by micro-scale structures. To analyze the micro mechanism (scattering caused by micro-scale structures) of the macro aero-optical phenomena (distorted images received by an optical sensor), a transient simulator for aero-optical effects (TSAO) is developed in this study. It is independent of the mesh resolution and can simulate the time-varying aero-optical phenomena through the mechanism of photon energy scattering. Because of the low computation cost, TSAO is suitable for analyzing aero-optical effects in the preliminary design stage of high-speed aircraft.

The artificial vortex structure is used in TSAO. Trolinger et al. proposed a method using randomly distributed gas spheres to supplement the CFD results, which effectively reduced the computation cost of aero-optical simulation [16,17]. In a previous study, we improved Trolinger’s method and designed a more accurate simulator that can simulate the directional dependence of aero-optical effects [9]. However, [9,16,17] used the operation of superposing multi-frame wavefront snapshots, which is equivalent to computing the steady-state distorted wavefront. As a result, they can only be used to analyze steady-state aero-optical effects.

In this paper, the artificial vortex structure method is expanded to the simulation of the transient aero-optical effects. The scaling model for distorted wavefronts from wind tunnel experiments is used to design the density distribution in the artificial vortex structure. As a result, both the temporal resolution required for transient simulation and the spatial resolution for simulating the scattering caused by micro-scale structures are satisfied. The density structure of the high-speed flow field changes with extremely high frequency, usually up to the magnitude of MHz [15]. In recent years, high-performance optical measuring equipment has been used to conduct high-frequency sampling of the disturbed light field through high-speed flow fields. Some models of the temporal and spatial evolution law of the transient distorted wavefront have been obtained. Wittich et al. proposed a normalization method for the spectrum of streamwise jitter ($\theta_{x}$) on the research basis of the scaling law for the root mean square of optical path difference (${\mathrm{OPD}}_{rms}$) in the boundary layer [18]. This normalization method makes the normalized frequency and spectrum amplitude independent of the flow conditions. The $\theta_{x}$ of the subsonic turbulent boundary layer measured in the wind tunnel experiments was used to verify the normalization method, and the results showed that the amplitudes of the spectra under different flow conditions were all overlapped to one curve after normalization, which indicated that a general scaling model exists to describe the transient aero-optical effects under different subsonic flow conditions. Subsequently, Gordeyev et al. found that after normalization, the $\theta_{x}$ of the supersonic turbulent boundary layer measured in wind tunnel experiments was coincident with that under subsonic conditions [19], which indicated that, under all flow conditions, streamwise aero-optical effects follow a similar scaling law. Gordeyev et al. found that the streamwise-distorted wavefront was pure convective [20], which means the evolution of $\theta_{x}$ has a spatio-temporal equivalence, indicating the Taylor frozen flow hypothesis is true. Based on the normalization method proposed by Wittich et al. [18], Gordeyev et al. [20] obtained the scaling law expression of the amplitude spectrum for $\theta_{x}$, which provides a theoretical basis for quantitatively describing the transient aero-optical effects caused by the turbulent boundary layer. Lucca et al. proposed a one-dimensional traveling harmonic model to describe the spatio-temporal evolution process of the streamwise distorted wavefront [21]. The traveling harmonic model can predict the change in the amplitude spectrum of $\theta_{x}$ under different sizes of observation aperture. Butler et al. then compared the wind tunnel data with the prediction of the one-dimensional traveling harmonic model for different aperture sizes, finding that their variation trends are consistent [22]. Through wind tunnel experiments, Kemnetz et al. verified the one-dimensional traveling harmonic model is also suitable for the forced shear layer [23].

The contribution of this study is that aero-optical effects are first analyzed in the aspect of scattering caused by micro-scale structures, and a transient simulation technique is developed for the micro analysis method. The proposed method describes the scattering mechanism of photon energy in the inhomogeneous medium, which does not rely on the assumption of local homogeneity compared with the current refraction explanation. Thus, it is more suitable for analyzing the aero-optical effects in the hypersonic flow field containing many micro-scale structures.

The remainder of this paper is organized as follows: In Section 2, the micro mechanism of aero-optical effects is presented. The design for TSAO is described in Section 3. The performance of TSAO in simulating the macro distortion through the proposed micro mechanism is verified in Section 4, accompanied by two examples for its application in the parameter optimization of the star sensor on a hypersonic vehicle. Finally, the conclusions are drawn in Section 5.

## 2. Aero-Optical Effects of Micro-Scale Structures

In this section, the explanation of the aero-optical distortion is explained in terms of the aspect of micro-scale structures. The result from the quantitative analysis of the propagation equation indicates that micro-scale structures may contribute more to image distortion than large-scale structures which are widely the concern of present research.

### 2.1. Micro Explanation for Macro Aero-Optical Phenomena

**Figure 1:**
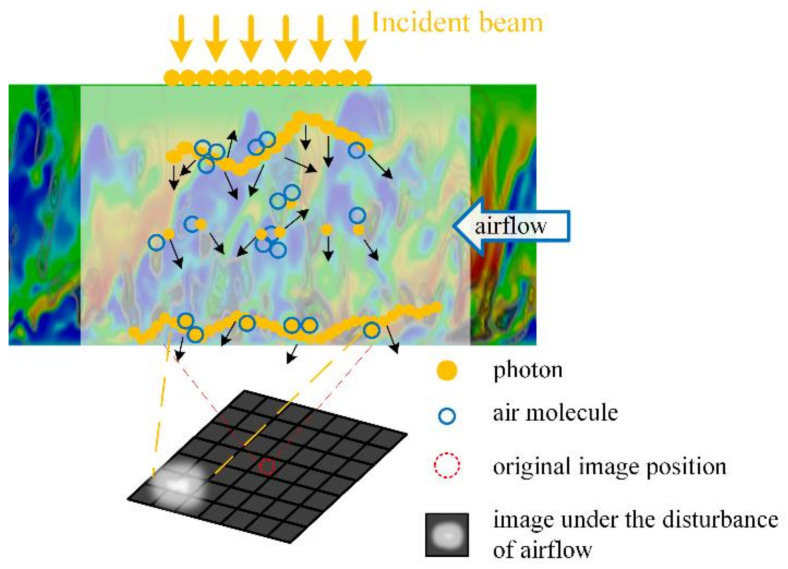
Disturbance in light transmission and the resulting distorted image.

The macro aero-optical phenomena observed on an optical sensor are usually deflection and blurring of the image, as shown at the bottom of Figure 1.

The deflection and blurring of the image can be explained by quantum optics, as shown in the upper part of Figure 1. The incident beam can be decomposed into many photons, and the density field is composed of airflow molecules. The energy of a photon is scattered by molecules, resulting in a scattering light field. Coherent superposition of different scattering light fields finally leads to the energy distribution received by the optical sensor. It is difficult to track each molecule in the simulation process. The usual approximate method involves describing the optical properties of all molecules in the local region with a parameter: the refractive index. This approximation method assumes that the optical properties of molecules in a local region are consistent, namely, uniform distribution. When there is much small-scale turbulence, the local homogeneity is no longer established; as a result, the refractive index defined based on the number density of molecules is no longer valid.

### 2.2. Micro Mechanism for Scattering of Photon Energy

The Helmholtz equation is usually used to describe the transmission of light in current aero-optical research [14], which is

(1) $$\nabla^{2}E+{\frac{\omega }{c_{0}{}^{2}}}^{2}n^{2}E=0,$$

where E is the vector of electric field, ω is the angular frequency of the light, $c_{0}$ is the light speed in a vacuum, and n is the refractive index, which varies with spatial position in inhomogeneous media, namely $n\left(x,y,z\right)$. However, Equation (1) is not accurate. The complete description for light transmission in inhomogeneous media is as follows [24]:

(2) $$\nabla^{2}E+{\frac{\omega }{c_{0}{}^{2}}}^{2}n^{2}E+2\nabla \left(E\cdot \nabla \right)\ln n=0$$

Equation (2) has an additional term containing the gradient of the refractive index compared with Equation (1). In the traditional view, the third term in Equation (2) contributes far less than the second term. However, when light transmits in micro-scale structures, the magnitude of the third term in Equation (2) cannot be ignored, and the ratio of the two terms satisfies [12]:

$$\frac{\left\|\nabla \left(E\cdot \nabla \right)\ln n\right\|}{\left\|{\omega^{2}/c_{0}}^{2}n^{2}E\right\|}=\frac{\lambda^{2}}{2\pi }C\int_{k_{0}}^{k_{i}}\left(k^{4}{\left(k^{2}+k_{0}{}^{2}\right)}^{-11/6}\exp\left(-\frac{k^{2}}{k_{i}{}^{2}}\right)\right)dk$$

where λ is the light wavelength; k=2π/Λ is the spatial frequency of turbulence, $k_{0}{=2\pi /\Lambda }_{0}$ and $k_{i}{=2\pi /\Lambda }_{i}$; Λ is the size of turbulence; $\Lambda_{0}$ and $\Lambda_{i}$ are outer and inner size, respectively; and C is the coefficient of the von Karman spectrum and satisfies

$${\left(C\int_{k_{0}}^{k_{i}}\left(k^{4}{\left(k^{2}+k_{0}{}^{2}\right)}^{-11/6}\exp\left(-\frac{k^{2}}{k_{i}{}^{2}}\right)\right)dk\right)}^{2}=1$$

**Figure 2:**
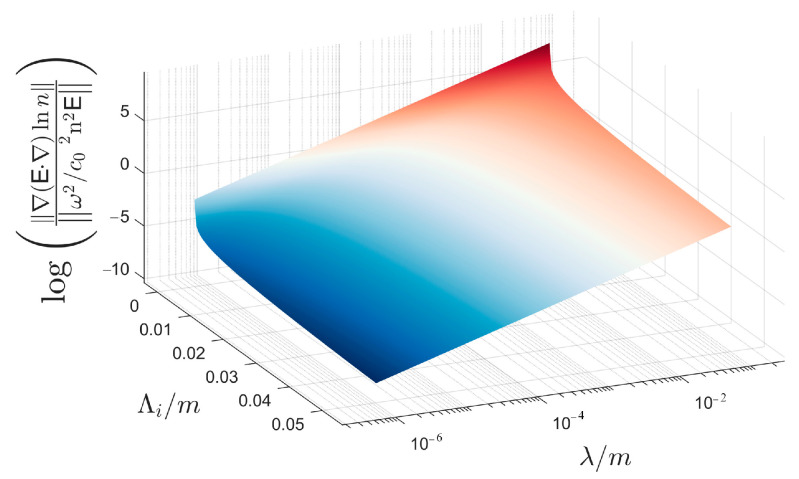
Variation trend in the ratio of the two terms in Equation (2) with wavelength and inner size.

As shown in Figure 2, the ratio of the two terms in Equation (2) increases exponentially with the decrease in $\Lambda_{i}$ and the increase in λ. Therefore, Equation (1) cannot accurately describe the transmission of long-wave light in micro-scale structures, whereas Equation (2) is a more universal form.

**Figure 3:**
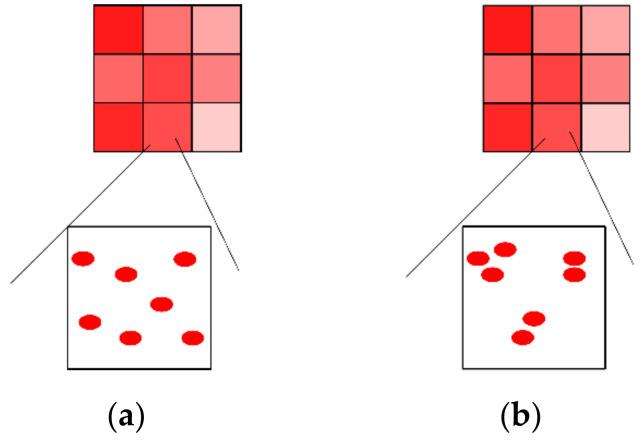
The refractive index distribution resolved by computational fluid dynamics (CFD) mesh and the actual distribution of air molecules: (**a**) Molecules are uniformly distributed in a grid; (**b**) molecules are nonuniformly distributed in a grid.

When using Equation (2) in aero-optical simulations, the difficulty is that the current mesh resolution of CFD cannot resolve the gradient of the refractive index. As shown in Figure 3, the grids with different transparencies represent the distribution of the refractive index that CFD mesh can resolve: the macro spatial density difference. When a grid is magnified, the distribution of air molecules can be observed, which is the micro-scale structure that the grid cannot resolve: the micro spatial density difference. The density difference is the main cause of aero-optical wavefront distortion [25]. CFD mesh can be applied to the case of uniform distribution, as shown in Figure 3a, but it will cause errors in the case shown in Figure 3b.

To analyze the influence of micro-scale structures on the incident light, we need a new aero-optical simulation technique. In Section 3, a transient simulator that can simulate the distorted wavefront caused by structures of all sizes without precision mesh is developed.

## 3. Design of the Transient Simulator for Aero-Optical Effects Caused by Micro-Scale Structures

**Figure 4:**
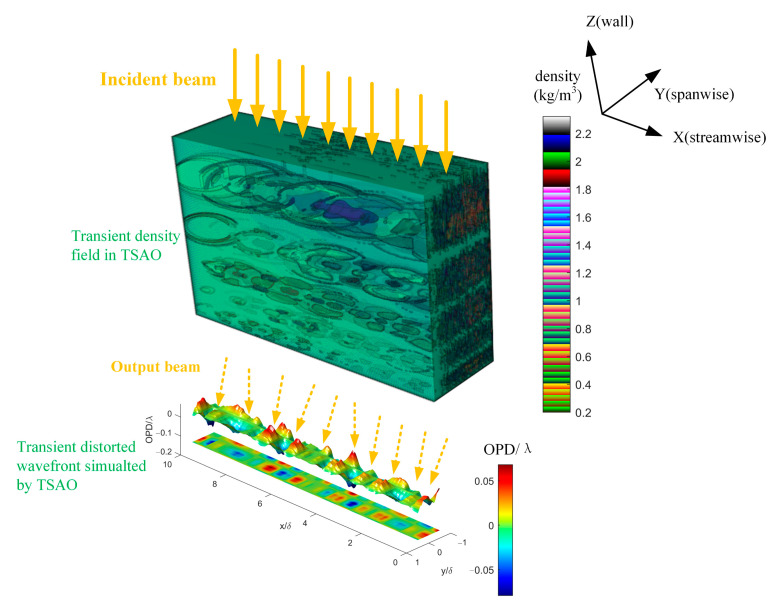
The configuration of the transient simulator for aero-optical effects (TSAO).

Molecules in the flow field are in high-speed movement. Large-scale structures are generally considered stable. The steady-state simulator [9] we designed, based on the steady-state hypothesis, simulates the aero-optical effects caused by large-scale structures. However, micro-scale structures are unsteady, so an aero-optical transient simulator (TSAO) is needed to simulate the high-dynamic distorted wavefront and the scattering process of the photon on its transmission path. There are two steps in the design of TSAO: transient distorted wavefront and transient density field, as shown in Figure 4. The transient distorted wavefront is designed by the scaling model. The transient density field is constructed by the artificial vortex structure.

### 3.1. Scaling Model of the Transient Distorted Wavefront in the TSAO

The spectrum of the distorted wavefront contains the energy information of the photons scattered by airflow molecules, so the scaling model for the spectrum is a design basis of the TSAO. The accuracy of the scaling model is not affected by the mesh resolution, which is critical to resolving the aero-optical effects caused by micro-scale structures with low computation cost.

#### 3.1.1. Spectral Characteristics of Transient Distorted Wavefront

At present, much attention has been paid to the characteristics of streamwise distortion. The normalized amplitude spectra of streamwise jitter caused by the subsonic and supersonic turbulent boundary layers have a similar scaling model [19,20]

(3) $${\hat{\theta }}_{x}\left(St_{\delta x}\right)={\hat{\theta }}_{peak}\frac{St_{\delta x}}{1+{\left(St_{\delta x}/0.75\right)}^{5/3}},$$

where $\theta_{x}$ is the streamwise jitter; $St_{\delta x}=\delta f/U_{\infty }$ is the normalized frequency in streamwise; the normalization eliminates the effects of different flow velocities $U_{\infty }$ and boundary layer thicknesses δ on the frequency f; $\left|{\hat{\theta }}_{peak}\right|$ is the peak value of the amplitude spectrum; and the normalized amplitude $\left|{\hat{\theta }}_{x}\left(St_{\delta x}\right)\right|$ is obtained from the amplitude spectrum of streamwise jitter $\left|\theta_{x}\left(f\right)\right|$ by the following normalization operation [18].

(4) $${\hat{\theta }}_{x}\left(St_{\delta }\right)=\frac{\theta_{x}\left(f\right)}{\frac{\rho_{\infty }}{\rho_{SL}}M^{2}\frac{\delta f_{s}}{U_{\infty }}},$$

where $\rho_{\infty }$ and $\rho_{SL}$ are the freestream density and reference density of sea level, respectively; M is the Mach number; and $f_{s}$ is the sampling frequency of $\theta_{x}$. The normalization in Equation (4) eliminates the effect of different freestream densities and Mach numbers. When combining both the normalizations in Equations (3) and (4), the scaling model in Equation (3) is suitable for all flow conditions. This provides the basis for the design of a transient simulator that can be used in a wide range of flow conditions.

The optical path difference (OPD) and $\theta_{x}$ have the following geometric relationship [18,20]:

(5) $$\theta_{x}\left(t\right)=-\frac{\partial \mathrm{OPD}\left(x,y,t\right)}{\partial x}$$

According to the differential properties of the Fourier transform and Equation (5), the amplitude spectra of streamwise OPD and $\theta_{x}$ satisfy

(6) $${\mathrm{OPD}}_{x}\left(f\right)=\frac{\theta_{x}\left(f\right)}{f},$$

where ${\mathrm{OPD}}_{x}$ represents the streamwise OPD.

According to Equations (3), (4) and (6), the scaling model for the amplitude spectrum of ${\mathrm{OPD}}_{x}$ can be written as

(7) $${\mathrm{OPD}}_{x}\left(St_{\delta x}\right)={\hat{\theta }}_{peak}\frac{1}{1+{\left(St_{\delta x}/0.75\right)}^{5/3}}M^{2}f_{s}\frac{\rho_{\infty }}{\rho_{SL}}{\left(\frac{\delta }{U_{\infty }}\right)}^{2}$$

To eliminate the influence of the differences in flow conditions, Equation (7) is normalized and we obtain the normalized scaling model for the amplitude spectrum of ${\mathrm{OPD}}_{x}$ as

(8) $${\hat{\mathrm{OPD}}}_{x}\left(St_{\delta x}\right)={\hat{\theta }}_{peak}\frac{1}{1+{\left(St_{\delta x}/0.75\right)}^{5/3}},$$

where ${\hat{\mathrm{OPD}}}_{x}\left(St_{\delta x}\right)={\mathrm{OPD}}_{x}\left(St_{\delta x}\right)/M^{2}f_{s}\frac{\rho_{\infty }}{\rho_{SL}}{\left(\frac{\delta }{U_{\infty }}\right)}^{2}$ is the normalized amplitude spectrum.

Since the OPD is related to the energy distribution on the image plane, using Equation (8) in TSAO can reflect the angle distribution in which photons are scattered away from the original direction. The low-frequency component is dominated by steady large-scale structures, while the high-frequency component corresponds to disturbances from dynamic micro-scale structures.

As for the spanwise OPD (${\mathrm{OPD}}_{y}$), since experiments showed that its convective velocity is zero [20], the spatial distribution of ${\mathrm{OPD}}_{y}$ is considered to be time-invariant. The spatial amplitude spectrum [20] is used to describe ${\mathrm{OPD}}_{y}$

(9) $${\mathrm{OPD}}_{y}\left(k_{y}\right)\propto \sqrt{\frac{1}{1+{\left(k_{y}/0.4\right)}^{8/3}}},$$

where $k_{y}$ is the spanwise spatial frequency.

#### 3.1.2. Transient Distorted Wavefront Construction Based on Traveling Harmonic Model

According to the principle of Fourier transform, the spectrum has a one-to-one correspondence with the signal in the time or space domain. However, the OPD caused by the turbulence has strong randomness. At present, the research on the OPD spectrum only finds significant characteristics of the amplitude spectrum, while the phase spectrum under different flow conditions cannot be described by a uniform scaling model. Therefore, the random phase is combined with the scaling model of the amplitude spectrum.

In streamwise, since ${\mathrm{OPD}}_{x}$ has spatio-temporal equivalence, the component of ${\mathrm{OPD}}_{x}$ at a certain frequency f can be described by the one-dimensional traveling harmonic model [21] as

(10) $$\mathrm{OPD}(x,t)=\sin\left(\frac{2\pi f}{U_{c}}x-2\pi ft\right),$$

where $U_{c}$ is the streamwise convective speed of OPD and $2\pi f/U_{c}=k_{x}$ is the streamwise spatial frequency. Thus, $k_{x}x$ represents the phase variation with the streamwise distance. 2πft represents the phase variation with time *t*.

To describe the scattering of photons caused by moving molecules, by superposing the harmonics of multiple frequencies, the traveling harmonic model in Equation (10) is used to generate the streamwise OPD caused by the density field at a certain wall height in a boundary layer (the wall height is defined as the distance from a certain position in the boundary layer to the wall surface)

(11) $$\begin{array}{c} {\bar{\mathrm{OPD}}}^{zk}\left(x,t\right)=\frac{\sum_{i=1}^{m_{k}}A_{xi}\sin\left(k_{xi}x-k_{xi}U_{c}t+\varphi_{x0i}\right)}{\sum_{i=1}^{n_{x}}A_{xi}}\\ \overset{sampling\;at\;t=N\Delta t}{=}\frac{\sum_{i=1}^{m_{k}}A_{x}{}_{i}\sin\left(\frac{2\pi }{\delta U_{c}/U_{\infty }}St_{\delta xi}x-N\frac{\delta U_{c}/U_{\infty }}{St^{s}}+\varphi_{x0i}\right)}{\sum_{i=1}^{n_{x}}A_{x}{}_{i}} \end{array}$$

where ${\bar{\mathrm{OPD}}}^{z_{k}}\left(x,t\right)$ represents the normalized OPD caused by the density field at the *k*’th wall height layer. The method to divide the wall height layer is described at the end of this subsection. $A_{x}{}_{i}$ is the normalized amplitude of the *i*th harmonic component, which is calculated by the scaling model in Equation (8). $St_{\delta xi}$ is the normalized frequency of the *i*th harmonic. The ratio of the streamwise convective velocity to the freestream velocity $U_{c}/U_{\infty }$ increases with the Mach number in the interval of 0.82~0.9 [19]. $St^{s}$ is the normalized sampling frequency of TSAO, which satisfies $St^{s}>2\max\left(St_{\delta xi}\right)$ according to the sampling theorem; *N* is the number of the sampling time step; $\varphi_{x0i}$ is the initial phase of the *i*th harmonic component, and it is selected as a random number within the interval of 0–2π to represent the randomness of the phase spectrum; and $m_{k}$ is the number of the superposed harmonics to generate ${\mathrm{OPD}}_{x}$ signals at the *k*th wall height layer. Since only a limited number of harmonics can be superposed in the TSAO code, we use the non-uniform sampling method [26] to select discrete frequency points to improve the fidelity of the generated OPD. $n_{x}$ is the total number of streamwise discrete frequency points satisfying $n_{x}=\sum_{k}m_{k}$.

As in streamwise, OPD in spanwise can also be described by superposed harmonics with the random phase. Since the convective speed of OPD in spanwise is zeros, the time-dependent term is not included in harmonics, namely

(12) $${\bar{\mathrm{OPD}}}_{}^{z_{k}}\left(y\right)=\frac{\sum_{j=1}^{n_{y}}A_{y}{}_{i}\sin\left(k_{yj}y+\varphi_{y0j}\right)}{\sum_{j=1}^{n_{y}}A_{y}{}_{j}}=\frac{\sum_{j=1}^{n_{y}}A_{y}{}_{j}\sin\left(\frac{2\pi }{\delta U_{c}/U_{\infty }}St_{\delta yj}y+\varphi_{y0j}\right)}{\sum_{j=1}^{n_{y}}A_{y}{}_{j}},$$

where the normalized spanwise amplitude $A_{y}{}_{j}$ is calculated by Equation (9). The meanings of other symbols are the same as in Equation (11).

By combining Equations (11) and (12), the expression of the two-dimensional OPD caused by the density field at a certain wall height layer is obtained as follows:

(13) $$\begin{array}{l} {\bar{\mathrm{OPD}}}_{}^{z_{k}}\left(x,y,t\right)=\\ \frac{\sum_{i=1}^{m_{k}}A_{x}{}_{i}\sin\left(\frac{2\pi }{\delta U_{c}/U_{\infty }}St_{\delta xi}x-N\frac{\delta U_{c}/U_{\infty }}{St^{s}}+\varphi_{x0i}\right)\sum_{j=1}^{n_{y}}A_{y}{}_{j}\sin\left(\frac{2\pi }{\delta U_{c}/U_{\infty }}St_{\delta yj}y+\varphi_{y0j}\right)}{\sum_{i=1}^{n_{x}}A_{x}{}_{i}\sum_{j=1}^{n_{y}}A_{y}{}_{j}} \end{array}$$

Equation (13) contains the scattering of photon energy caused by density structures of all sizes, which can be used to simulate the macro aero-optical phenomena caused by the micro mechanism.

Since density structures with different sizes contribute differently to the spectrum of the OPD, OPD signals should be generated according to the spectra of different wall height layers. However, current scaling models can only provide the OPD spectral characteristics caused by the superposed disturbances of density fields at different wall height layers. Therefore, we manually set the relation between the wall height layer and the corresponding frequencies of the OPD signals in TSAO as: the larger the structure size (the higher the wall height [9]), the lower the OPD frequency. As such, each frequency in the OPD spectrum corresponds to a certain size of density structure (at a certain wall height). The density field construction in Section 3.2 can be achieved. If a finer scaling model is developed in the future, the current rule can be replaced without changing the other settings in the TSAO.

### 3.2. Density Field Construction Based on Layered-Ellipse Vortex Model

The second step for TSAO design is the transient density field. We propose a new artificial vortex structure, the layered-ellipse model, to construct the two-dimensional density field, which can reflect disturbances from structures of all sizes without a precision mesh. Finally, the method to simulate the directional dependence of aero-optical effects (beam with different incident angles) and the method to calibrate the TSAO are presented.

#### 3.2.1. Density Field Construction

**(a) Layered-ellipse vortex model**

**Figure 5:**
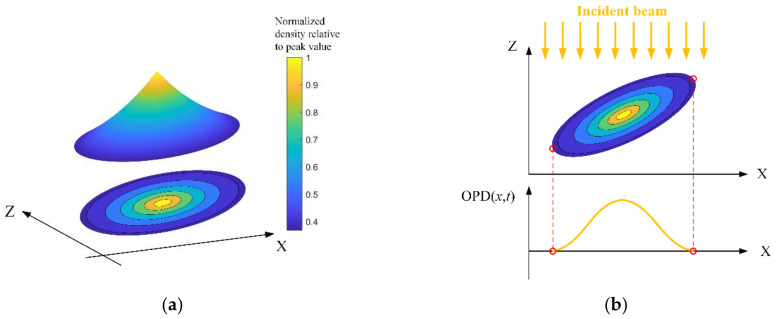
Layered-ellipse vortex model (X, streamwise; Z, wall direction): (**a**) Surface and projection of the layered-ellipse vortex model in two-dimensional density field; (**b**) parallel beam transmits through a layered-ellipse vortex and produces the optical path difference (OPD).

In our previous steady-state simulator, the gas ellipsoid model with uniform internal density was used to approximate the density structures in the boundary layer [9]. The directional dependence of aero-optical effects can be simulated by designing the tilt angle and oblateness of the gas ellipsoid. In the TSAO, we change the setting of the uniform internal density to simulate the spectral characteristics of the OPD caused by density structures of different sizes. Density distribution in a real vortex is usually presented as a layered structure. Starting from a vortex core, the density varies along with the radial direction layer by layer, but the density variation inside a layer is small (see Figure 23 in [10] and Figure 8 in [27]). The layered shape can exactly reflect disturbances from density structures of different sizes, which is abstracted as the layered-ellipse vortex model, as shown in Figure 5a.

The turbulent structures in a supersonic boundary layer are quite complex, such as the hairpin vortex, horseshoe vortex, and so on. The proposed layered-ellipse vortex model only improves the accuracy of simulating the optical property of the turbulent structure compared with the previous artificial vortex structure [9,16,17]; simulating the other properties of the turbulent structure is not the focus of TSAO.

Since the layered-ellipse vortex model is symmetric, when the parallel beam transmits through it, the produced OPD is also symmetric, as is shown in Figure 5b. At the two endpoints of the vortex model (red circles in Figure 5b), the geometrical distance of the light path inside the ellipse is zero, resulting in the singularity of the OPD at endpoints. In other words, the OPD at the endpoint cannot be changed by changing the density distribution along the radial direction of the ellipse, which limits the presentation ability of the layered-ellipse vortex model.

**Figure 6:**
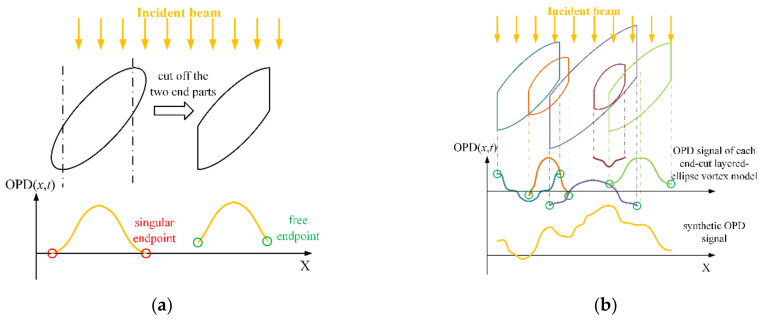
The end-cut layered-ellipse vortex model: (**a**) We cut off the two end parts of the layered-ellipse vortex model; (**b**) the OPD signal produced by the density field at a certain wall height layer is decomposed to the OPDs of multiple concatenated end-cut layered-ellipse vortex models (The colors of end-cut layered-ellipse show that they cause different OPDs. The bottom OPD signal plotted by the yellow curve is the synthesis of the OPDs with different colors above it).

The OPD signal generated by Equation (13) is random. To construct the corresponding density field by the layered-ellipse vortex model, we cut the two end parts of the layered-ellipse vortex model, as shown in Figure 6a. After cutting off the two end parts, the geometric distance of the light path inside the ellipse is not zero. As a result, the original singular endpoints where the OPD is fixed value become free endpoints. As such, by changing the density distribution along the radial direction, an end-cut layered-ellipse vortex model can represent the density field corresponding to any symmetric OPD signal.

As shown in Figure 6b, the streamwise OPD signal ${\mathrm{OPD}}_{}^{z_{k}}\left(x,t_{0}\right)$, which is caused by a certain wall height layer ($z=z_{k}$) at a certain moment ($t=t_{0}$), can always be decomposed into the following form

(14) $${\mathrm{OPD}}_{}^{z_{k}}\left(x,t_{0}\right){=\mathrm{OPD}}_{}^{z_{k}}\left({\left[x\right]}^{1},t_{0}\right)+{\mathrm{OPD}}_{}^{z_{k}}\left({\left[x\right]}^{2},t_{0}\right)+\cdots +{\mathrm{OPD}}_{}^{z_{k}}\left({\left[x\right]}^{n},t_{0}\right)$$

where ${\left[x\right]}^{i}$, i=1,2,⋯,n, is the range of the *i*th end-cut layered-ellipse vortex model concatenated streamwise.

Equation (14) indicates that the end-cut layered-ellipse vortex model, which can only generate symmetric OPD signals, can generate arbitrary random OPD signals after concatenation. To ensure the uniqueness of the decomposition result in Equation (14), we set the position of the two adjacent vortex models streamwise to satisfy ${\left[x\right]}_{l}^{i}={\left[x\right]}_{m}^{i-1}$, indicating the left end of the downstream vortex model ${\left[x\right]}_{l}^{i}$ is aligned with the center of the upstream vortex model ${\left[x\right]}_{m}^{i-1}$. According to the following steps, the decomposition results in Equation (14) can be uniquely determined

(15) $$\begin{array}{l} \mathrm{Initial}:\;{\left\lfloor \mathrm{OPD}\right\rfloor }_{}^{z_{k}}\left(x,t_{0}\right){=\mathrm{OPD}}_{x}^{z_{k}}\left(x,t_{0}\right)\\ {\mathrm{step}1:\;\mathrm{OPD}}_{}^{z_{k}}\left({\left[x\right]}_{l}^{i}\to {\left[x\right]}_{m}^{i},t_{0}\right)={\left\lfloor \mathrm{OPD}\right\rfloor }_{}^{z_{k}}\left({\left[x\right]}_{l}^{i}\to {\left[x\right]}_{m}^{i},t_{0}\right)\\ {\mathrm{step}2:\;\mathrm{OPD}}_{}^{z_{k}}\left({\left[x\right]}_{m}^{i}\to {\left[x\right]}_{r}^{i},t_{0}\right)={\mathrm{OPD}}_{}^{z_{k}}\left({\left[x\right]}_{l}^{i}\to {\left[x\right]}_{m}^{i},t_{0}\right)\\ {\mathrm{step}3:\;\mathrm{OPD}}_{}^{z_{k}}\left({\left[x\right]}^{i},t_{0}\right)={\mathrm{OPD}}_{}^{z_{k}}\left({\left[x\right]}_{l}^{i}\to {\left[x\right]}_{m}^{i}\to {\left[x\right]}_{r}^{i},t_{0}\right)\\ \mathrm{step}4:\;{\left\lfloor \mathrm{OPD}\right\rfloor }_{}^{z_{k}}\left(x,t_{0}\right)={\left\lfloor \mathrm{OPD}\right\rfloor }_{}^{z_{k}}\left(x,t_{0}\right)-{\mathrm{OPD}}_{}^{z_{k}}\left({\left[x\right]}^{i},t_{0}\right),i=i+1 \end{array}$$

where ${\left\lfloor \mathrm{OPD}\right\rfloor }_{}^{z_{k}}\left(x,t_{0}\right)$ is the current remaining OPD signal to be decomposed, ${\left[x\right]}_{l}^{i}\to {\left[x\right]}_{m}^{i}$ represents the streamwise coordinates from the left endpoint to the center of the *i*th vortex model, and the subscript *r* represents the right endpoint. Step2 indicates that the OPD signal generated by a vortex model is symmetric. Step3 connects the left and right halves of a the vortex model into a complete one.

The construction method of density field described in this subsection is for the two-dimensional streamwise density field. Since designing the vortex model in the three-dimensional density field needs to deal with the coupling between streamwise and spanwise, it is an open problem in the current aero-optical research. Therefore, the method to construct the three-dimensional density fields in TSAO involves arranging multiple streamwise two-dimensional density fields along spanwise, as shown in Section 4.1.

**(b) Determine density field in vortex model**

To simulate the scattering of the photon on its transmission path, we need to determine the density structure $f\left(x,z\right)$ inside the vortex model, which satisfies

(16) $${\mathrm{OPD}}_{}^{z_{k}}\left({\left[x\right]}^{i}\right){=K}_{\mathrm{GD}}\int_{z_{LB}}^{z_{UB}}\left[f\left(x,z\right)+R\left(x,z\right)\right]dz,$$

where $K_{\mathrm{GD}}$ is the Gladstone-Dale constant; $R\left(x,z,t_{0}\right)$ is the residual part of the density field except for the layered-ellipse structure; $z_{LB}$ and $z_{UB}$ are the lower and upper bound of the vortex model in the wall direction (Z), respectively.

According to geometry, the expression of the density distribution in the layered-ellipse model is

(17) $$f\left(x,z\right)=f\left(\sqrt{\frac{{\left[x\cos\left(\theta \right)+z\sin\left(\theta \right)\right]}^{2}}{a^{2}}+\frac{{\left[-x\sin\left(\theta \right)+z\cos\left(\theta \right)\right]}^{2}}{b^{2}}}\right)=f\left(\phi \right),$$

where $\phi =\sqrt{\frac{{\left[x\cos\left(\theta \right)+z\sin\left(\theta \right)\right]}^{2}}{a^{2}}+\frac{{\left[-x\sin\left(\theta \right)+z\cos\left(\theta \right)\right]}^{2}}{b^{2}}}$ represents tilted ellipses with different sizes; *a*, *b*, and θ are the semi-major axis, semi-minor axis, and tilt angle of the ellipse, respectively. The three parameters are evaluated in the same way as in [9] to simulate the directional dependence of aero-optical effects. If we constrain $x\in \left[{\left[x\right]}_{l}^{i},{\left[x\right]}_{r}^{i}\right]$ and $\left({\left[x\right]}_{l}^{i}-{\left[x\right]}_{r}^{i}\right)<a$, then Equation (17) is for the end-cut layered-ellipse model.

We use the polynomial basis function to approximate the $f\left(x,z\right)=f\left(\phi \right)$ in Equation (17) as

(18) $$f\left(x,z\right)=f\left(\phi \right)=c_{0}+c_{1}\phi +c_{2}\phi^{2}+\cdots +c_{n}\phi^{n}+O\left(\phi^{n}\right),$$

where $c_{0}$, $c_{1}$, ⋯, $c_{n}$ are the polynomial coefficients to be solved and $O\left(\phi^{n}\right)$ is the remainder term.

Substitute Equation (18) into Equation (16) and use the least square method to solve the following overdetermined equations

(19) $$\left[\begin{array}{cccc} \int_{z_{LB}}^{z_{UB}}dz & \int_{z_{LB}}^{z_{UB}}\phi dz & \cdots & \int_{z_{LB}}^{z_{UB}}\phi^{n}dz \end{array}\right]\left[\begin{array}{c} c_{0}\\ c_{1}\\ \vdots \\ c_{n} \end{array}\right]=\frac{{\mathrm{OPD}}_{}^{z_{k}}\left({\left[x\right]}^{i},t_{0}\right)}{K_{\mathrm{GD}}},$$

Then we obtain the polynomial coefficients in Equation (18). Since the analytic expression of the coefficient matrix in Equation (19) is extremely complex, we use the numerical integration method to calculate $\int_{z_{LB}}^{z_{UB}}\phi^{i}dz$, i=1,2,⋯,n. The highest order of the approximation polynomial in Equation (18) is constrained to *n* = 6 to accelerate the code execution speed of the TSAO.

Substitute Equation (19) into Equation (16) and obtain the residual density field $R\left(x,z\right)$ as

(20) $$\int_{z_{LB}}^{z_{UB}}R\left(x,z\right)dz=\frac{{\mathrm{OPD}}_{}^{z_{k}}\left({\left[x\right]}^{i}\right)}{K_{\mathrm{GD}}}-\int_{z_{LB}}^{z_{UB}}\left(c_{0}+c_{1}\phi +c_{2}\phi^{2}+\cdots +c_{n}\phi^{n}\right)dz$$

Since the residual density field represents the remaining parts of the total density field except for the layered-ellipse structure, it has no definite spatial shape. For the convenience of computation, we assume $R\left(x,z\right)$ is uniform along the wall height direction, namely $R\left(x,z\right)=R\left(x\right)$. Substitute it into Equation (20) and we obtain

(21) $$R\left(x,z\right)=R\left(x\right)=\frac{{\mathrm{OPD}}_{}^{z_{k}}\left({\left[x\right]}^{i}\right)/K_{\mathrm{GD}}-\int_{z_{LB}}^{z_{UB}}\left(c_{0}+c_{1}\phi +c_{2}\phi^{2}+\cdots +c_{n}\phi^{n}\right)dz}{z_{UB}-z_{LB}}$$

Thus, the density field in TSAO can be determined using Equations (16), (18), (19) and (21). Since density structures that cause all distortions are contained in this density field, it can be used to describe the process of the scattering the photon on its transmission path by air molecules. Although some constraints (such as the ideal layered-ellipse shape of the density structure) are introduced, they are the abstract of optical properties for the irregular turbulence. The numerical example in Section 4.2 proves that the constraint of the ideal ellipse shape for the vortex model has little influence on the dominant structure of the density field.

#### 3.2.2. TSAO Calibration Method

**(a) Directional dependence of aero-optical effects**

The directional dependence of aero-optical effects has been widely confirmed by experiments and simulations [10,20,28,29]. The method of constructing the density field presented in Section 3.2.1 is for the case of the normal incident beam in Figure 6b. A general method to treat the case of any incident direction is provided in this subsection.

**Figure 7:**
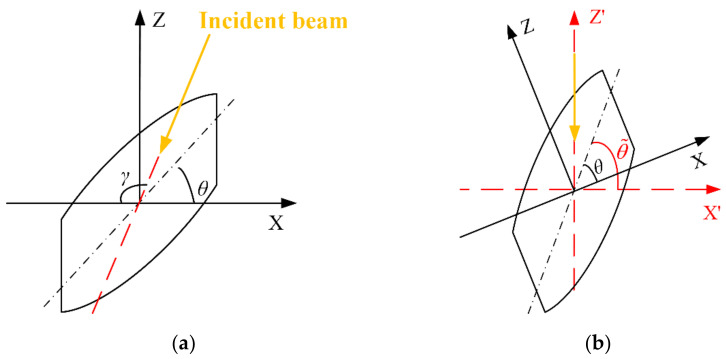
Method to deal with the beam incident from any direction: (**a**) Beam incident from any direction; (**b**) any direction is equivalent to the normal incident beam after rotation.

As shown in Figure 7a, when the beam is incident at any angle $\gamma \in \left(0,\pi \right)$ to the vortex model whose tilt angle is θ, rotate the vortex model to the tilt angle of

(22) $$\tilde{\theta }=\gamma +\theta -\frac{\pi }{2},$$

as shown in Figure 7b. When the beam is normally incident to the rotated vortex model whose tilted angle is $\tilde{\theta }$, the light path is the same as that in Figure 7a. Therefore, in the rotated coordinate frame $X^{\prime }—Z^{\prime }$ of Figure 7b, the method in Section 3.1 can be used to determine the density field under the condition of arbitrary incidence direction. The only modification is to replace the original tilt angle θ with the equivalent tilted angle $\tilde{\theta }$ in Equation (22).

After the equivalent transformation of incident direction, to simulate the directional dependence of aero-optical effects, Equation (13) needs to be corrected by the incident angle γ as follows:

(23) $$\begin{array}{l} {\bar{\mathrm{OPD}}}_{}^{z_{k}}\left(x,y,t\right)=\\ h\left(\gamma \right)\frac{\sum_{i=1}^{m_{k}}A_{x}{}_{i}\sin\left(\frac{2\pi }{\delta U_{c}/U_{\infty }}St_{\delta xi}x-N\frac{\delta U_{c}/U_{\infty }}{St^{s}}+\varphi_{x0i}\right)\sum_{j=1}^{n_{y}}A_{y}{}_{j}\sin\left(\frac{2\pi }{\delta U_{c}/U_{\infty }}St_{\delta yj}y+\varphi_{y0j}\right)}{\sum_{i=1}^{n_{x}}A_{x}{}_{i}\sum_{j=1}^{n_{y}}A_{y}{}_{j}} \end{array}$$

where the correction factor $h\left(\gamma \right)$ is related to the incident angle γ. The shape of the function curve of $h\left(\gamma \right)$ is similar to a quadratic function with an upward opening and the minimum is at $\gamma =\frac{\pi }{2}-\theta$ (see Figure 8 in [9]).

**(b) TSAO Output calibration**

Since the OPD signal at each wall height layer ${\bar{\mathrm{OPD}}}_{}^{z_{k}}\left(x,y,t\right)$ generated by Equation (23) is in the normalized form, the transient OPD output from TSAO is also in the normalized form, which satisfies

(24) $$\begin{array}{l} \bar{\mathrm{OPD}}\left(x,y,t\right)=\sum_{k=1}^{k_{\max}}{\bar{\mathrm{OPD}}}_{}^{z_{k}}\left(x,y,t\right)\\ =\sum_{k=1}^{k_{\max}}h\left(\gamma \right)\frac{\sum_{i=1}^{m_{k}}A_{x}{}_{i}\sin\left(\frac{2\pi }{\delta U_{c}/U_{\infty }}St_{\delta xi}x-N\frac{\delta U_{c}/U_{\infty }}{St^{s}}+\varphi_{x0i}\right)\sum_{j=1}^{n_{y}}A_{y}{}_{j}\sin\left(\frac{2\pi }{\delta U_{c}/U_{\infty }}St_{\delta yj}y+\varphi_{y0j}\right)}{\sum_{i=1}^{n_{x}}A_{x}{}_{i}\sum_{j=1}^{n_{y}}A_{y}{}_{j}}\leq 1 \end{array}$$

Equation (24) cannot directly describe the distortion under a given flow condition. To match the TSAO output with the real distortion, a calibration parameter needs to be introduced. As the design of the previous steady-state simulator [9], we use ${\mathrm{OPD}}_{rms}$ as the criterion for the output calibration of the simulator. Through calibration, the ${\mathrm{OPD}}_{rms}$ of TSAO is ensured to be consistent with the boundary layer aero-optical effects prediction model (BLPM) [20], namely

(25) $$rms\left[\kappa \bar{\mathrm{OPD}}\left(x,y,t\right)\right]{=\mathrm{OPD}}_{rms}^{p},$$

where κ is the calibration parameter to be solved; ${\mathrm{OPD}}_{rms}^{p}$ is the predicted ${\mathrm{OPD}}_{rms}$ of the BLPM under the given flow condition, which satisfies [20]

$${\mathrm{OPD}}_{rms}^{p}{=\mathrm{BK}}_{\mathrm{GD}}\rho_{\infty }M^{2}\delta \sqrt{C_{f}}G\left(M\right),$$

where B is a constant. $G\left(M\right)$ is an empirical function of the Mach number, and $\sqrt{C_{f}}$ denotes the compressible skin friction.

According to the linear property of the root mean square

(26) $$rms\left[\kappa \bar{\mathrm{OPD}}\left(x,y,t\right)\right]=\kappa \cdot rms\left[\bar{\mathrm{OPD}}\left(x,y,t\right)\right]$$

Since OPD is spatio-temporal equivalent in streamwise (X) and time-invariant in spanwise (Y) according to Section 3.1, then Equation (26) can be rewritten as

(27) $$rms\left[\kappa \bar{\mathrm{OPD}}\left(x,y,t\right)\right]=\kappa \cdot rms\left[\bar{\mathrm{OPD}}\left(x_{Ap},y,t\right)\right]=\kappa \cdot rms\left[\bar{\mathrm{OPD}}\left(x_{T},y\right)\right],$$

where $x_{Ap}$ is the range of the observed aperture in streamwise and $x_{T}$ represents the total length of the streamwise OPD that flows through the aperture range at the convective velocity $U_{c}$ within the simulation time.

Equation (27) indicates that the time-domain root mean square of the transient OPD in the aperture can be calculated using the OPD from a wider spatial range on the upstream side. Therefore, during the OPD generation stage, set $x=x_{T}$ in Equation (24) and calculate the root mean square of the OPD in the $x_{T}$ range. Then, we obtain $rms\left[\bar{\mathrm{OPD}}\left(x_{Ap},y,t\right)\right]=rms\left[\bar{\mathrm{OPD}}\left(x_{T},y\right)\right]$ in Equation (27). Substitute this into Equation (25) and the calibration parameter κ can be solved

$$\kappa =\frac{{\mathrm{BK}}_{\mathrm{GD}}\rho_{\infty }M^{2}\delta \sqrt{C_{f}}G\left(M\right)}{rms\left[\bar{\mathrm{OPD}}\left(x_{T},y\right)\right]}$$

Multiply Equation (23) by the calibration parameter κ, and the method for generating real OPD signals of each wall height layer is obtained

(28) $$\begin{array}{l} {\mathrm{OPD}}_{}^{z_{k}}\left(x,y,t\right)=\\ h\left(\gamma \right)\kappa \frac{\sum_{i=1}^{m_{k}}A_{x}{}_{i}\sin\left(\frac{2\pi }{\delta U_{c}/U_{\infty }}St_{\delta xi}x-N\frac{\delta U_{c}/U_{\infty }}{St^{s}}+\varphi_{x0i}\right)\sum_{j=1}^{n_{y}}A_{y}{}_{j}\sin\left(\frac{2\pi }{\delta U_{c}/U_{\infty }}St_{\delta yj}y+\varphi_{y0j}\right)}{\sum_{i=1}^{n_{x}}A_{x}{}_{i}\sum_{j=1}^{n_{y}}A_{y}{}_{j}} \end{array}$$

**Figure 8:**
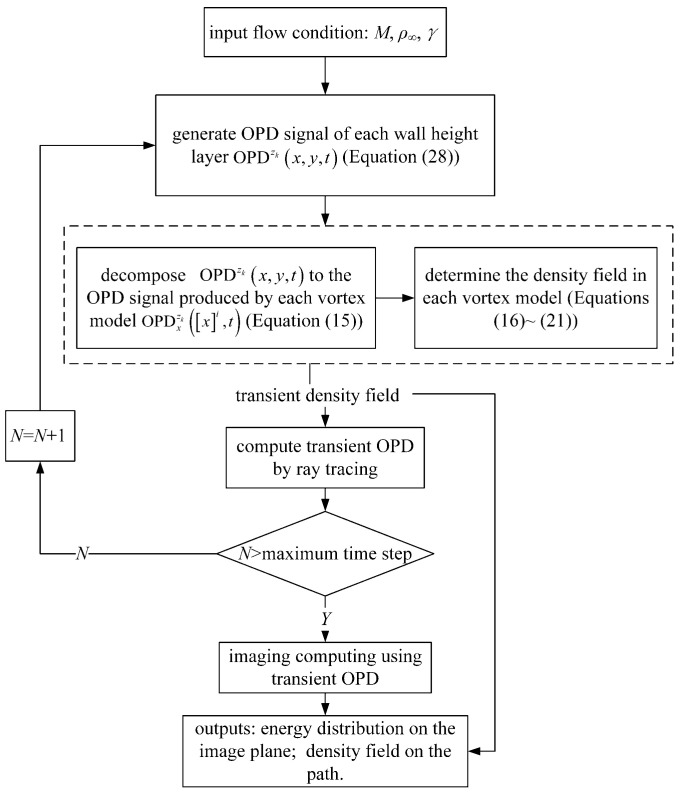
The running process of the TSAO.

Now, we have described the complete design of TSAO, and its running process is shown in Figure 8.

## 4. Results and Discussion

In this section, we first present examples to verify the performance of the TSAO for simulating the aero-optical effects caused by micro-scale structures. Then two examples of TSAO application to analyzing the optimal parameters of the star sensor (aperture size and exposure time) on the hypersonic vehicle are described, where the parallel beam with a wavelength of λ of 550 nm is used to simulate starlight.

### 4.1. TSAO Simulates Aero-Optical Phenomena Based on Micro Mechanism

In the first example, we use TSAO to simulate the distorted wavefront and density field in a given flow condition. The results show that correct aero-optical phenomena can be obtained by the micro mechanism of photon energy scattering.

#### 4.1.1. Simulation of Distorted Wavefront and Density Field in a Given Flow Condition

**Figure 9:**
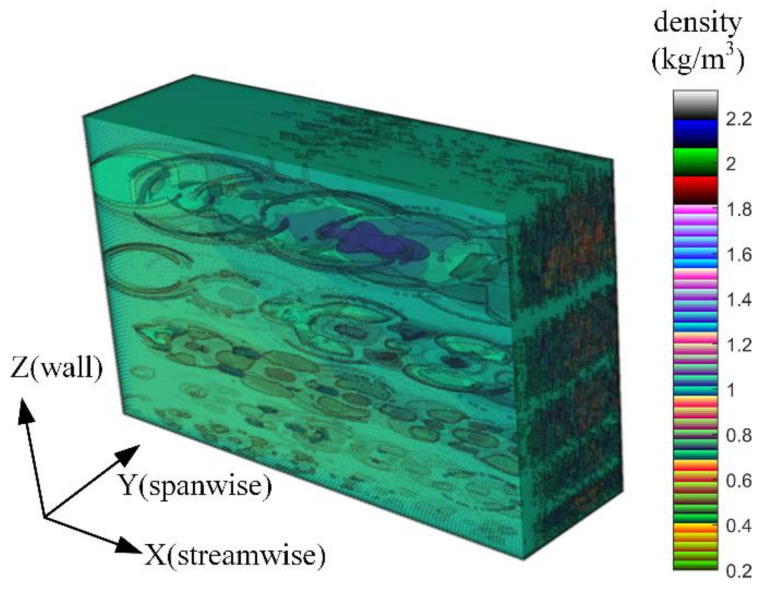
The three-dimensional density field in TSAO.

Figure 9 displays the three-dimensional density field obtained through arranging multiple two-dimensional streamwise density fields constructed by the method in Section 3.2; the corresponding height (*H*) and Mach number (*M*) are listed in Table 1. The relation between height *H* and freestream density $\rho_{\infty }$ is described by an approximate atmospheric density model of $\rho_{\infty }=1.5373e^{-0.1462H}$, where the units for $\rho_{\infty }$ and *H* are $\mathrm{kg}/m^{3}$ and km, respectively. $\rho_{\infty }$ affects the output of the TSAO through the calibration parameter κ in Equation (28). The density field is divided into five wall height layers, and each layer corresponds to a certain range of structure sizes. The relation between the structure size and the wall height is described by Equation (1) in [9].

In Figure 9, the streamwise density field is the expected layered-ellipse shape, and the size of the density structure increases with the wall height, which is consistent with current research conclusions [10,30]. Since the spanwise density field is composed of many two-dimensional density fields, it has no specific shape. The five wall height layers are stacked one by one and there is no overlap between the layers. Whether or not the density fields of each wall height layer overlap does not affect the simulation accuracy of TSAO. Since OPD is accumulated layer by layer in the light transmission process according to Equation (16), TSAO can output correct results as long as the disturbance caused by each wall height layer is accumulated in the calculation of the ray tracing.

**Figure 10:**
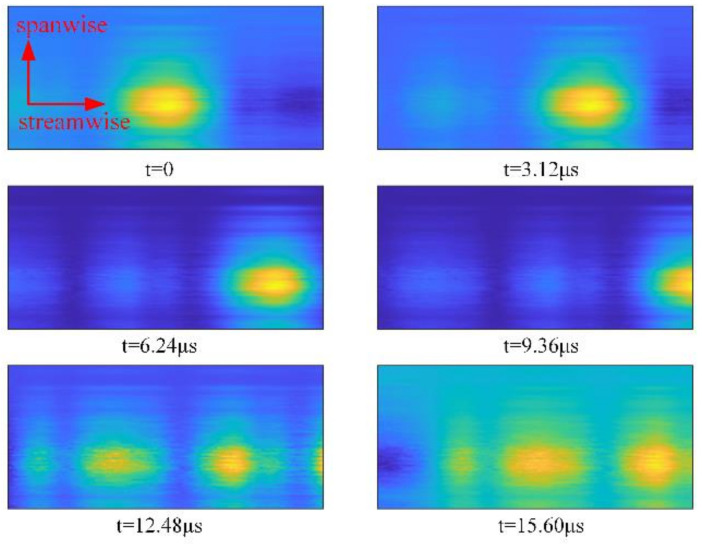
Transient OPDs at different times.

Figure 10 shows the two-dimensional OPD produced when the incident beam transmits through the three-dimensional density field in Figure 9. Transient OPDs in the aperture at different times are provided. The maximum normalized frequency in streamwise $\max\left(St_{\delta x}\right)$, sampling frequency $f_{s}$, and aperture size are listed in Table 1.

In Figure 10, the feature structure of the transient OPD moves downstream with time, reflecting the time-varying characteristic of streamwise OPD. The feature structure has no obvious movement spanwise because the spatial spectrum scaling model used in the spanwise design is time-invariant.

**Figure 11:**
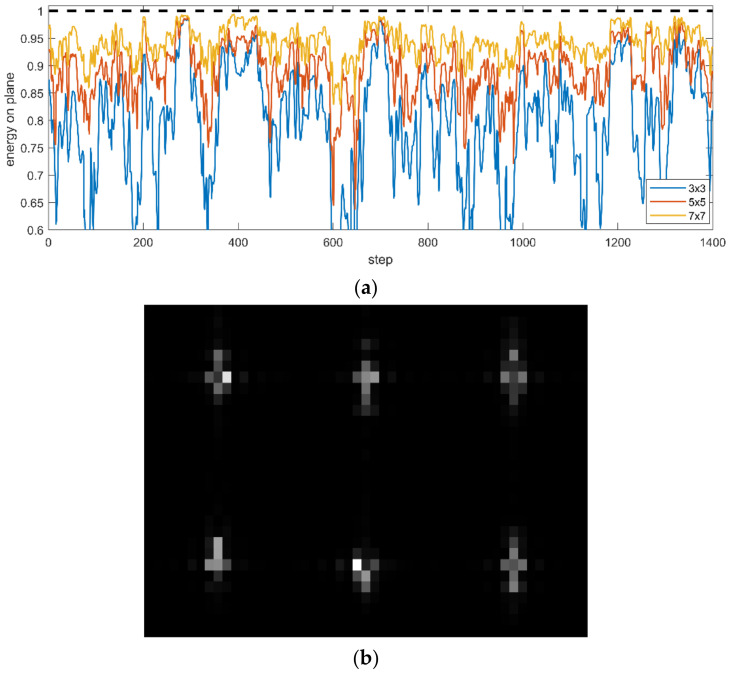
Simulation results of photon energy scattering: (**a**) Energy proportion in different ranges; (**b**) image blurring caused by photon energy scattering.

Figure 11a shows the energy distribution on the image plane corresponding to the transient OPDs in Figure 10, where the dotted line represents the total energy in the entire image plane and the solid line with different colors represents the energy proportion within the range of 3 × 3, 5 × 5 and 7 × 7 pixels near the peak value.

Figure 11a indicates that the energy received at the center of the image plane changes dynamically with time, reflecting the scattering effect caused by the high-speed air molecules on the photon energy, which corresponds to the moving feature structure of the OPD in Figure 10. The energy proportion in Figure 11a increases with the increase in the statistical range because the scattering effect causes photons to deviate from the original direction, and the resulting macro phenomenon is image blurring, as shown in Figure 11b.

#### 4.1.2. Contribution of Various-Sized Density Structures to Distorted Wavefront

**Figure 12:**
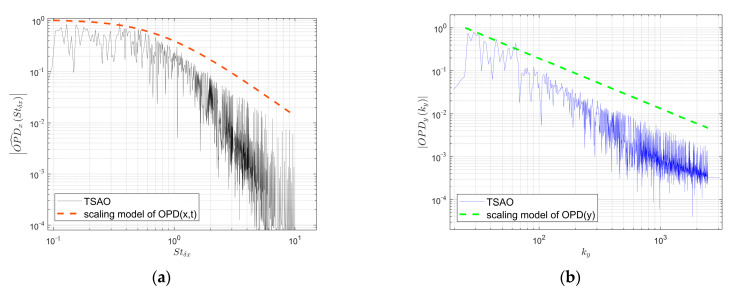
Amplitude spectra of the OPD from TSAO: (**a**) Streamwise; (**b**) spanwise.

The amplitude spectra of the local OPD (the OPD in the range of one pixel in Figure 10) from TSAO are plotted in Figure 12, where Figure 12a is the streamwise time spectrum and Figure 12b is the spanwise spatial spectrum. The reason for amplitude chattering is that the TSAO code can only simulate limited frequency points, so the amplitude at frequency points that are not simulated reduces sharply. This is an inherent phenomenon when simulating continuous spectra using computer code. In Figure 12, the amplitude spectra are consistent with the scaling models in Equations (8) and (9), indicating that the distorted wavefront simulated by TSAO (Figure 10) contains the influence from micro-scale structures.

**Figure 13:**
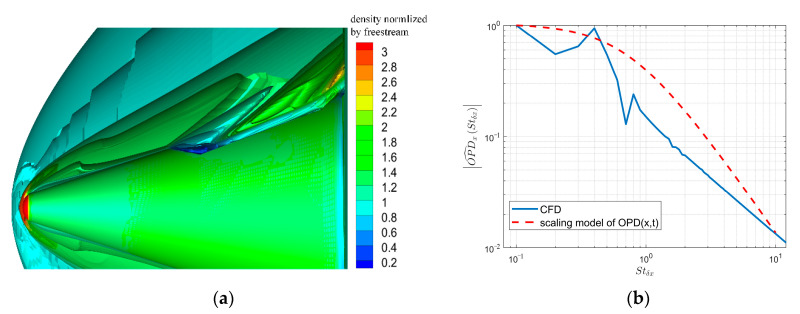
Simulation result with CFD mesh: (**a**) Isosurface of density field; (**b**) amplitude spectra of OPD.

As a comparison, Figure 13 shows the OPD spectrum from a CFD density field whose flow condition is *M* = 3, *H* = 20 km, and the mesh resolution is 2 mm. Compared with TSAO, the OPD spectrum calculated from the CFD mesh has an obvious shortage in the middle- and high-frequency components, which is caused by the mesh resolution no capturing disturbances from micro-scale structures. According to Section 2, with the increase in light wavelength and Reynolds number of the flow (high Reynolds number leads to small inner size), the error becomes more obvious.

**Figure 14:**
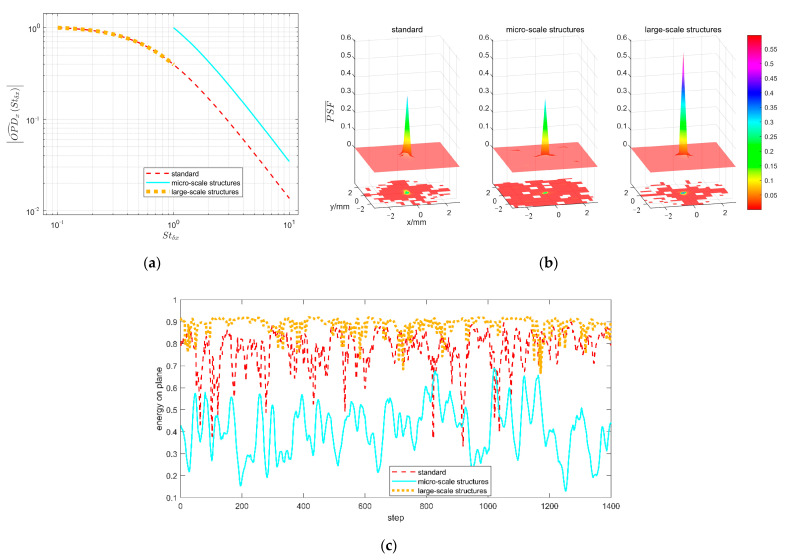
Effects of different-sized structures on the point spread function (PSF): (**a**) Amplitude spectra of streamwise OPD; (**b**) PSF and its projection; (**c**) energy proportion within the range of 5 × 5 pixels near the peak value of the PSF.

Figure 14 shows the simulation result of the point spread function (PSF) caused by different-sized density structures. The effect of micro-scale structures is reflected in the high-frequency OPD, while the effect of large-scale structures is reflected in the low-frequency OPD. Three different amplitude spectra of streamwise OPD are used as the scaling model in TSAO, as shown in Figure 14a, where the standard model is Equation (8). The amplitude spectra for micro- and large-scale structures are the high- and low-frequency parts of the standard model.

Figure 14b shows transient PSFs corresponding to OPDs caused by different-sized structures, where the PSF is normalized by the total energy on the image plane as follows

$$\bar{\mathrm{PSF}}\left(x,y,t\right)=\frac{\mathrm{PSF}\left(x,y,t\right)}{\int \mathrm{PSF}\left(x,y,t\right)dxdy},$$

The projection in Figure 14b is the part satisfying $\bar{\mathrm{PSF}}\left(x,y,t\right)$ > 1 × 10^−5^, where a larger turbulent structure leads to more energy concentrating near the peak value of the PSF. Figure 14c shows the energy proportion in the range of 5 × 5 pixels near the peak value of the PSF at different sampling times. The relation between the energy distribution and the structure scale found in Figure 14b is suitable for all moments. The reason for this trend is the medium with a size close to or smaller than the wavelength of the incident light causing a large scattering angle compared with a large-scale medium according to the optical principle.

The examples given in this section indicate that TSAO can simulate all frequency components of the distorted wavefront, which is critical for analyzing the aero-optical effects caused by micro-scale structures.

### 4.2. Verification of Layered-Ellipse Vortex Model Based on Proper Orthogonal Decomposition Method

The layered-ellipse vortex model in TSAO is used to reflect disturbances from density structures of different sizes. We use the proper orthogonal decomposition (POD) [31] to decompose the density field in the TSAO. The result is analyzed to verify the reasonability of the vortex model.

**Figure 15:**
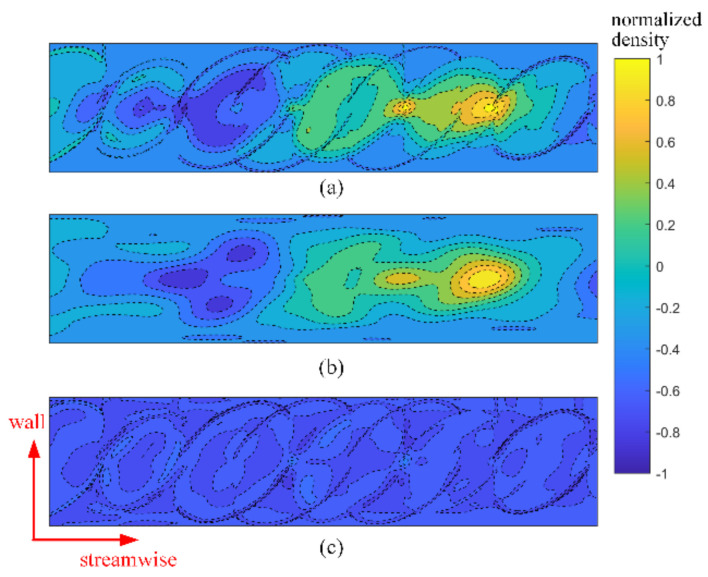
The transient density field and the reconstructed density field with proper orthogonal decomposition (POD) modes (density field is normalized to [−1, 1] by the minimum and maximum value): (**a**) Transient density field; (**b**) reconstructed density field with 10 POD modes (96.7%); (**c**) residual density field.

**Figure 16:**
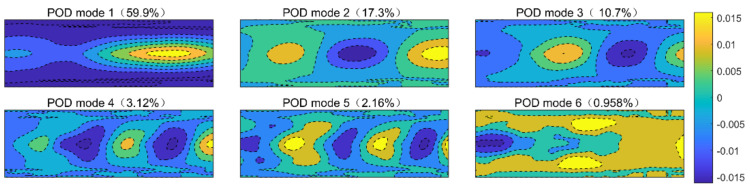
POD modes of the density field.

Since the density field of each wall height layer is constructed in the same method in the TSAO, only the density field of a single wall height layer is decomposed. Figure 15a is a transient density field constructed by the method in Section 3.2. Figure 16 depicts the first six POD modes with the highest energy of the density field in Figure 15a, where the energy is defined as the eigenvalue of the corresponding POD mode, and the energy proportion (the ratio of the eigenvalue of a POD mode to the sum of all eigenvalues) is shown in the parentheses of each subfigure’s caption. Differences in modes are reflected in the size of the density structure, indicating that the density field constructed by the layered-ellipse vortex model contains the density structures of different sizes. This vortex model is independent of the mesh resolution.

Figure 15b,c are the density field reconstructed with the first 10 POD modes and the residual density field. The reconstructed density field contains most of the energy, which has the characteristic of a large-scale layered density structure. The residual density field in Figure 15c mainly contains the boundary of the regular ellipse and internal density fluctuation with low energy. By comparing Figure 15b,c, the boundary of the layered-ellipse vortex model contains little energy of the density field, and the ideal boundary is exactly the constraint introduced in TSAO for computing convenience, indicating that the ideal boundary constraint has little influence on the accuracy of the density field.

In conclusion, the results of POD show that the layered-ellipse vortex model contains density structures of different sizes. It is a more accurate optical model for aero-optical simulation compared with current artificial vortex structures [7,14,15].

### 4.3. Applying TSAO to Analyzing the Aperture Effects of a Star Sensor

The aperture effects of aero-optics have important influences on the imaging quality of the finite aperture. In this experiment, we use TSAO to evaluate the imaging quality of optical sensors with different aperture sizes. The results can be used to choose the optimal parameters of the star senor to suppress the aero-optical effects.

**Figure 17:**
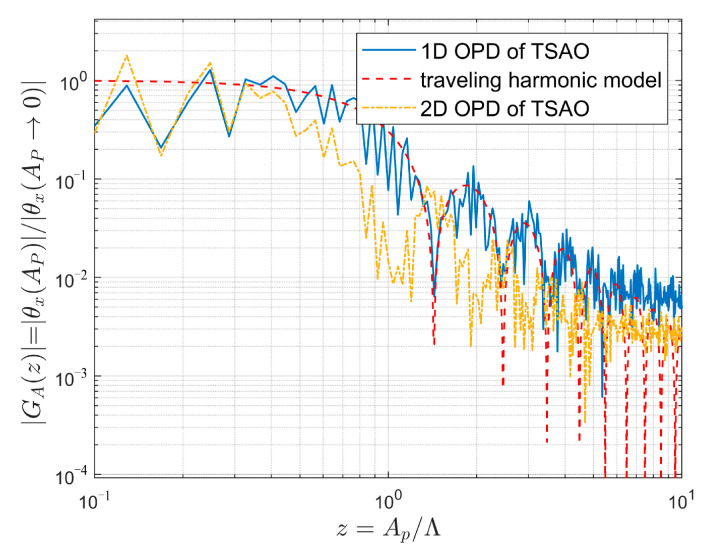
The aperture effects of $\theta_{x}$.

We calculate the influence of $z=A_{p}/\Lambda$ (ratio of the aperture size $A_{p}$ to the spatial wavelength in streamwise Λ) on $\left|\theta_{x}\right|$ (streamwise jitter amplitude) using the transient streamwise OPD (one-dimensional) from TSAO (the relation between OPD and $\theta_{x}$ is given by Equation (5)). The results are plotted using the solid blue line in Figure 17. The *x*-axis $z=A_{p}/\Lambda$ reflects the relative size of the aperture $A_{p}$ to the wavelength of a certain frequency harmonic $\Lambda =2\pi /k_{x}$ in the streamwise OPD. The *y*-axis $\left|G_{A}\left(z\right)\right|$ is the ratio of the streamwise jitter amplitude $\left|\theta_{x}\left(A_{P}\right)\right|$ = $\frac{\left|\theta_{x}\left(A_{P}\right)\right|}{\left|\theta_{x}\left(A_{P}\to 0\right)\right|}$ calculated by the OPD in the aperture to the streamwise local jitter amplitude of a single point $\left|\theta_{x}\left(A_{P}\to 0\right)\right|$, which reflects the scaling effect of the aperture size on $\theta_{x}$.

The red dashed line in Figure 17 is the transfer function of the aperture effects of $\theta_{x}$ obtained using the one-dimensional traveling harmonic model, which satisfies [21]:

$$G_{A}\left(z\right)=\frac{3\left[\sin\left(\pi z\right)-\left(\pi z\right)\cos\left(\pi z\right)\right]}{{\left(\pi z\right)}^{3}}$$

Figure 17 shows that the aperture effects of $\theta_{x}$ calculated by the one-dimensional OPD from TSAO are consistent with the theoretical model, and the variation trend is that with the increase in the relative aperture z, $\left|G_{A}\left(z\right)\right|$ tends to decay on the whole, but oscillates locally. The yellow dot-dash line in Figure 17 is $\left|G_{A}\left(z\right)\right|$, which is calculated using the two-dimensional OPD from TSAO. With the increase in the relative aperture z, $\left|G_{A}\left(z\right)\right|$ for the two-dimensional OPD gradually deviates from that of the one-dimensional traveling harmonic model, which was also found in [22] when analyzing data from wind tunnel experiments. The provided explanation was that the two-dimensional OPD is non-uniform in spanwise.

According to Equation (28), the amplitude of spanwise OPD varies with the spanwise coordinate (Y). Therefore, the $\theta_{x}$ calculated by two-dimensional OPD is actually the average of $\theta_{x}$ calculated by one-dimensional OPD with different spanwise coordinates. Since $\theta_{x}$ calculated by one-dimensional OPD can be positive or negative, the average effect of positive and negative values in the aperture range decreases the amplitude of $\theta_{x}$ at all frequency points. The spanwise spatial wavelength at the low frequency is larger than the aperture size, so the averaged $\theta_{x}$ in the aperture range is close to the local $\theta_{x}$. Conversely, the wavelength at high frequency is much smaller than the aperture size, and the averaged $\theta_{x}$ deviates more from the local $\theta_{x}$. This is the reason for the deviation of the yellow dot-dash line in Figure 17. However, the deviation direction found in [22] is opposite to that in Figure 17, indicating that $\left|G_{A}\left(z\right)\right|$ calculated by the two-dimensional OPD in [22] decays slower than that of the one-dimensional traveling harmonic model. However, no detailed explanation was given except for the non-uniformity of spanwise OPD. Since the coupling mechanism between the streamwise and spanwise remains unclear, the open problem remains of constructing the aperture effects model of $\theta_{x}$ corresponding to the two-dimensional OPD.

According to the principle of diffraction imaging, the complex amplitude of the light field on the image plane $U\left(\frac{x^{\prime }}{\lambda f},\frac{y^{\prime }}{\lambda f}\right)$ can be obtained by Fourier transform of the complex amplitude of the light field in the observation aperture $w\left(x,y\right)$

(29) $$\begin{array}{l} U\left(\frac{x^{\prime }}{\lambda f},\frac{y^{\prime }}{\lambda f}\right)=\iint w\left(x,y\right)e^{-j\frac{2\pi }{\lambda f}\left(xx^{\prime }+yy^{\prime }\right)}dxdy\\ w\left(x,y\right)=a\cdot e^{j\frac{2\pi }{\lambda }\mathrm{OPD}\left(x,y\right)} \end{array}$$

where $x^{\prime }$ and $y^{\prime }$ are the coordinates on the image plane, x and y are the coordinates in the observation aperture, $f_{L}$ is the focal length, and a is the amplitude of the incident light. Decompose OPD to the blurring component and the jitter components [14] as follows:

(30) $$\mathrm{OPD}\left(x,y\right){=\mathrm{OPD}}_{h}\left(x,y\right)+A\cdot x+B\cdot y,$$

where A⋅x and B⋅y are jitter components in streamwise and spanwise, respectively, satisfying $\theta_{x}=\arctan\left(A\right)$ and $\theta_{y}=\arctan\left(B\right)$. *A* and *B* can be solved by fitting the OPD surface using a plane A⋅x+B⋅y+C=0 through the least squares method. A⋅x+B⋅y is also the linear term of Zernike polynomials. ${\mathrm{OPD}}_{h}\left(x,y\right)$ is the blurring component.

Substituting Equation (30) into Equation (29) and using the phase shift properties of the Fourier transform, the total light field can be represented by the light field of the blurring component

(31) $$\begin{array}{c} U\left(\frac{x^{\prime }}{\lambda f_{L}},\frac{y^{\prime }}{\lambda f_{L}}\right)=\iint w_{h}\left(x,y\right)e^{j\frac{2\pi \left(A\cdot x+B\cdot y\right)}{\lambda }}e^{-j\frac{2\pi }{\lambda f_{L}}\left(xx^{\prime }+yy^{\prime }\right)}dxdy\\ =U_{h}\left(\frac{x^{\prime }}{\lambda f_{L}}-\frac{A}{\lambda },\frac{y^{\prime }}{\lambda f_{L}}-\frac{B}{\lambda }\right) \end{array}$$

where $w_{h}$ and $U_{h}$ are the light field of blurring component on the aperture and image plane, respectively.

**Figure 18:**
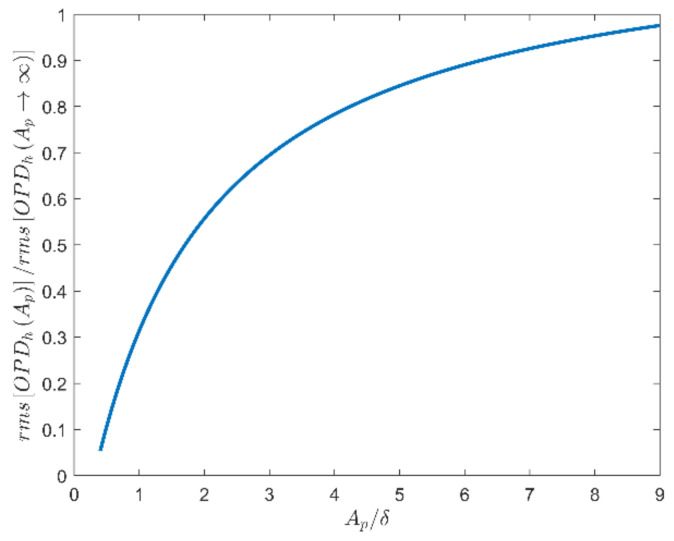
The aperture effects of the blurring component in the OPD.

Equation (31) indicates that the jitter component is the direct factor affecting the overall deviation of the navigation star’s position on the image plane in the background of celestial navigation, which is equivalent to adding a uniform position offset to all pixels. Therefore, suppressing the jitter can directly improve the accuracy of the star sensor. According to Figure 17, we select a large observation aperture to reduce the measurement error caused by $\theta_{x}$. However, the blurring component ${\mathrm{OPD}}_{h}\left(x,y\right)$ is also influenced by the aperture size. The root mean square of ${\mathrm{OPD}}_{h}\left(x,y\right)$ increases with the aperture size [20,32,33], as shown in Figure 18, which indicates that increasing the aperture size increases image blurring. Star map blurring reduces the accuracy of star centroid acquisition and leads to measurement error, as does the jitter component. To summarize, the aperture effects of the jitter and blurring components are contradictory. We found a tradeoff is necessary between the two kinds of effects through many aero-optical simulations to determine the optimal aperture size of the star sensor.

### 4.4. Applying TSAO to Analyzing the Influence of Exposure Time on Received Energy Distribution

The energy distribution on the image plane is a key index affecting the accuracy of celestial navigation. Because of the high dynamic characteristics of turbulence, it produces different disturbances in the incident light field at every moment, which lead to the superposition of blurring within the exposure time ($T_{e}$) of the star sensor. In this example, we use TSAO to study the relation of $T_{e}$ and energy distribution on the image plane (evaluated by the Strehl Ratio (SR)).

**Figure 19:**
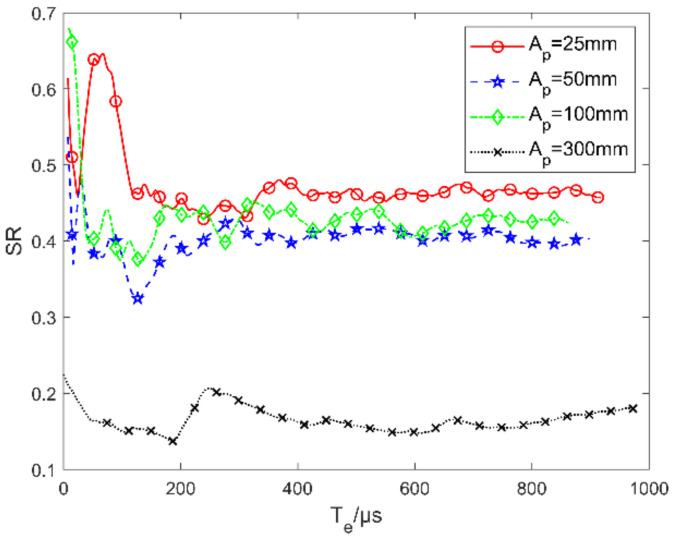
Variation of Strehl ratio (SR) with exposure time ($T_{e}$) and aperture size.

Under the flow condition given in Table 1, the SRs for different $T_{e}$ are plotted in Figure 19, where the results of different aperture sizes are also provided. The typical aperture size of a star sensor on a satellite at present is 25 mm; 50 and 100 mm mean doubling the aperture size constantly; other multiples of 25 mm can also be taken. $A_{p}$ = 300 mm corresponds to 10 multiples of the boundary layer thicknesses δ = 30 mm in TSAO. According to Figure 18, when $A_{p}/\delta$ > 10, $rms\left({\mathrm{OPD}}_{h}\right)$ reaches its maximum value. Thus, $A_{p}$ = 300 mm represents the condition of the maximum blurring component.

In Figure 19, SR tends to be stable with the increase in $T_{e}$, which agrees with the experimental observation in [34]. By comparing the plots of different aperture sizes, we find the speed to the steady state of SR increases with the aperture size. The reason is as follows: The jitter causes the overall deviation in the navigation star’s position on the image plane. With the increase in $T_{e}$, the energy distributions at different moments superpose, which leads to image blurring. The blurring caused by jitter tends to saturation (SR reaches the steady state) with the increase in $T_{e}$. According to Figure 17, the larger the aperture, the smaller the jitter, and a shorter $T_{e}$ is needed to reach the blurring saturation or the steady-state of SR.

The difference in steady-state SR with different aperture sizes is the result of both the aperture effects of jitter and blurring components. In Figure 19, the steady-state SR does not decrease monotonically with the increase in aperture size, where the non-monotonic steady-state SR appears in the case of $A_{p}$ < 100 mm. This is because the aperture effects of the jitter components and the blurring component have opposite trends with the aperture size, as shown in Figure 17 and Figure 18. In Figure 19, the steady-state SR is about 0.15 when $A_{p}$ = 300 mm, which coincides with the predicted value ${\mathrm{SR}}^{p}$ = 0.148 obtained by BLPM and the Maréchal approximation [9]. This indicates that the OPD output from the TSAO is accurate.

According to the above analysis, under the ideal condition, choosing an aperture $A_{p}$ = 25 mm and short exposure time ($T_{e}$ < 100 μs) is conducive to obtaining a clear star map. However, when considering the performance of an actual optical sensor, the configuration of $A_{p}$ = 25 mm and $T_{e}$ < 100 μs is not conducive to energy reception. As a result, the image quality is more susceptible to noise. Therefore, the optimal noise suppression and aero-optical effects suppression cannot be achieved simultaneously, so a tradeoff should be chosen according to the photosensitive performance of the star sensor and using many of aero-optical simulations under the flight state.

## 5. Conclusions

A new method for analyzing aero-optical effects caused by micro-scale structures is developed in this paper. The result from the quantitative analysis of the propagation equation indicates that micro-scale structures may contribute more than large-scale structures, which are the focus of present research. Since current aero-optical simulation methods cannot resolve micro-scale structures—for example, the CFD method is limited by mesh resolution [14,35] and the current artificial vortex structure method [9,16,17] has no available vortex model for micro-scale structures—an aero-optical transient simulator (TSAO) is designed to simulate the macro aero-optical phenomena through the micro mechanism of photon energy scattering. TSAO is independent of mesh resolution and has a low computation cost, so it is suitable for analyzing aero-optical effects in the preliminary design stage of high-speed aircraft. We provide examples of using TSAO to analyze the optimal aperture size and exposure time of a star sensor on a hypersonic vehicle. The results show that the obtained optimal parameters for suppressing aero-optical effects decrease the light flux. Therefore, a design based on a trade-off between the actual photosensitive performance of a star sensor and the aero-optical effects under the flight condition is needed.

By comparing feature structures on the OPD surface in Figure 4 and the OPD data in Figure 7 of [36], which was obtained in wind tunnel experiments, we found that the most significant difference is that the OPD simulated by TSAO has simple feature structures, whereas the real feature structures of OPD have irregular shapes whose streamwise length is larger than in spanwise overall. The reason for this difference is that the scaling model for the transient distorted wavefront in TSAO is independent in streamwise and spanwise aspects, whereas the feature structures in Figure 7 of [36] with various irregular shapes are due to the coupling mechanism between streamwise and spanwise, which is an open problem in current aero-optical research. To improve the performance of the TSAO, the coupling scaling model and coupling vortex model will be focuses of future work.

## Figures and Tables

**Table 1:** The flow condition and simulation parameters.

Parameter	Value	Parameter	Value
*H*	5 km	$\max\left(St_{\delta x}\right)$	10
*M*	5	$f_{s}$	16 MHz
$U_{\infty }$	1603 m/s	Aperture	25 mm (X) × 12.5 mm (Y)

## Data Availability

Data sharing not applicable.
